# The barriers and facilitators to the design and delivery of sustainable healthcare across NHS Scotland: A forcefield analysis

**DOI:** 10.1016/j.fhj.2024.100135

**Published:** 2024-04-21

**Authors:** Alice Harpur, Kirsty Crowe, Emily Turner, Katherine Jobling, Luis Loureiro Harrison, Gary Paul, Richard Tran, Patrick Farrell

**Affiliations:** Scottish Clinical Leadership Fellowship Programme, NHS Education for Scotland, 102 West Port, Edinburgh, EH39DN, United Kingdom

**Keywords:** Climate change, Sustainable healthcare, Forcefield analysis

## Abstract

Healthcare systems around the world have set ambitious targets to design and deliver more environmentally sustainable healthcare. To achieve these targets, individuals, teams, organisations, and whole systems will have to change current attitudes, practices and processes. Change management theory advocates for early identification of the influencing forces to change, so that actions can be taken to overcome the barriers and strengthen the facilitators to increase the likelihood of success. This project undertook a forcefield analysis exercise to identify the barriers and facilitators to the design and delivery of sustainable healthcare in Scotland. The exercise identified 12 facilitators and 12 barriers to sustainable change and formulated ten recommendations to strengthen the former and overcome the latter. It is hoped that the results will raise awareness of the factors that influence the design and delivery of sustainable healthcare and will inform what actions can be taken to increase the likelihood of success.

## Introduction

The triple planetary crisis of climate change, biodiversity loss, and pollution poses a significant threat to healthcare access and delivery. In 2022, UK summer heatwaves led to mass cancellations of elective work, with one fifth of hospitals forced to cancel operations during the 3 days of highest temperatures.^1^ Meanwhile, the adverse effects of the planetary crisis on human health, through issues such as air pollution, extreme weather events, mass population displacement, and changing distributions of disease vectors, are placing new and increasing demands on already over-stretched healthcare systems.^2^

Provision of healthcare, however, is also contributing to the problem and thus undermining its goal of improving health and wellbeing. It has been estimated that if healthcare was a single country, it would be the world's fifth largest emitter, accounting for 4.6% of greenhouse gas emissions.^3^

In response, there have been growing international efforts to deliver healthcare in a more environmentally sustainable way. At the World Health Organization's COP26 event, 50 countries committed to prioritising the development of environmentally sustainable healthcare systems.^4^ The United Kingdom (UK) was among these, and, since then, each of the devolved nations has made progress towards fulfilling the commitment. In 2022, the Scottish Government released its ‘NHS Scotland Climate Emergency and Sustainability Strategy’ which outlines actions NHS Scotland will take to achieve net-zero greenhouse gas emissions by 2040.^5^

To reach this goal, NHS Scotland will have to make significant changes to how it designs and delivers healthcare. To increase the likelihood of success, change management theory advocates for the early identification of influencers of change to enable the acquisition of appropriate knowledge and skills to overcome barriers and optimise facilitators. To our knowledge, no literature has articulated the barriers and facilitators to the design and delivery of environmentally sustainable healthcare within the NHS; this may represent a missed opportunity for NHS Scotland, and similar healthcare organisations, to pre-empt and act upon the barriers and facilitators that may make sustainability efforts less or more likely to succeed, respectively.

The primary aim of this study was to identify the barriers and facilitators to the design and delivery of sustainable healthcare within NHS Scotland. The secondary aim was to formulate a set of recommendations that would support progression towards net-zero.

## Method

### Study design

A forcefield analysis exercise was undertaken using the methodology first described by Kurt Lewin.^6^ Five steps were followed:

1) Propose a change
2) Identify barriers to the desired change
3) Identify facilitators to the desired change
4) Assign a numerical weight to each force to demonstrate its anticipated effect on the desired change
5) Consider what action can be taken to weaken the barriers and/or strengthen the facilitators

In step 1, the project team formulated a change state of ‘the design and delivery of sustainable healthcare across NHS Scotland’. The methodology of steps 2-5 is outlined below.

### Sample

Purposive sampling was used to recruit individuals actively involved in the design and/or delivery of sustainable healthcare in NHS Scotland. To achieve a broad perspective, three groups of individuals were approached: those undertaking work to design and/or deliver sustainable healthcare at an individual or team level; an organisational level; and a systems level. Interviewees were initially identified using the authors’ knowledge of national leaders and experts in sustainable healthcare accrued while working at national, regional and local levels on this subject. Further interviewees were identified from ‘snowball’ sampling of the original participants. Sampling continued until it was felt that no new barriers or facilitators were identified, and that thematic saturation was reached. Interviewees were from a variety of professional backgrounds including Medical (Primary and Secondary Care), Nursing, Pharmacy, Organisational Development and Quality Improvement, Undergraduate and Postgraduate Medical Education, and included individuals working in Clinical Sustainability roles within local and national healthcare organisations. Participants provided their personal reflections, rather than necessarily presenting the official position of their organisation, and spoke on the condition of anonymity.

### Data collection

Individuals were invited via e-mail to participate in a 1-hour semi-structured interview on MS-Teams. A topic guide was used to guide discussion, but interviewers remained flexible to explore new topics as they emerged. Seven team members participated in interviews, with either one or two members interviewing each participant. Field notes were taken during the conversations. To mitigate against the impact that interviewers' biases and opinions would have on the results, the barriers and facilitators identified were listed back to participants at the end of interviews so that they could confirm accuracy and offer any amendments.

### Data analysis

Upon conclusion of the interviews, team members compiled a list of the barriers and facilitators identified. Any duplicates were removed. Thematic analysis was used to classify the barriers and facilitators into key themes. The field notes were used to provide additional context and to facilitate team members reaching consensus on the identified themes.

### Generation of weighting and recommendations

The barriers and facilitators were independently scored from 0 to 5 by eight team members and a mean score was calculated. A score of 0 equated to a barrier or facilitator that was perceived to have no impact on influencing change, and a score of 5 equated to a barrier or facilitator that was perceived to have a very high impact on influencing change.

Through rounds of group discussions, consensus was reached on a list of ten recommendations that were felt to address each of these themes.

## Results

### Study sample

Twenty individuals from 12 healthcare organisations who were involved in the planning and/or delivery of healthcare services across NHS Scotland were interviewed. Interviewees represented a range of professional groups including Medical and Surgical Doctors, Public Health specialists, Nurses, Pharmacists, and Healthcare Managers. Interviewees held varying levels of seniority and represented both community- and hospital-based healthcare settings.

### The barriers to the design and delivery of sustainable healthcare

**Fig. 1:**
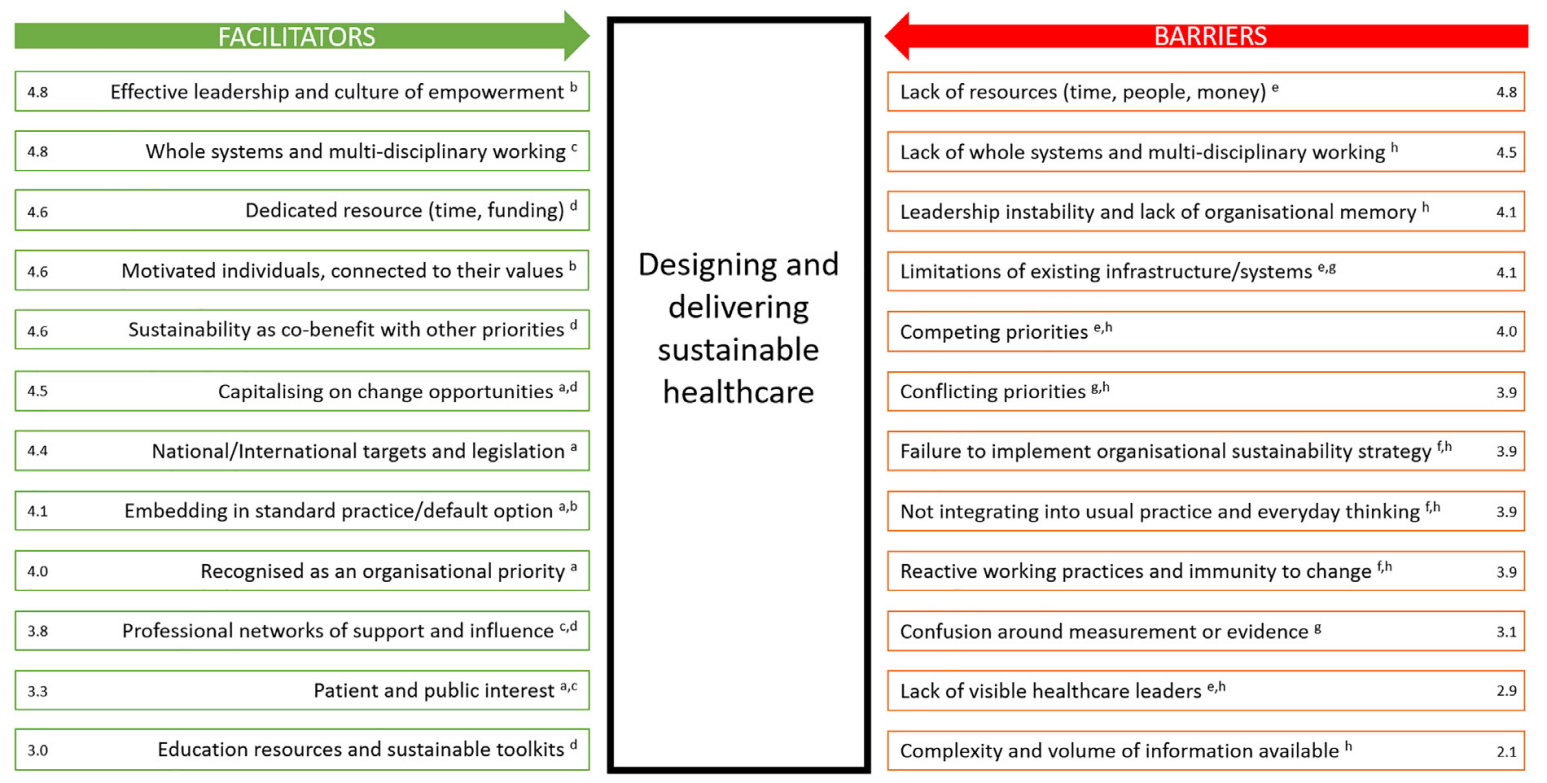
Forcefield analysis of barriers and facilitators to the design and delivery of sustainable healthcare. Numbers refer to weight of each force, scored from 0 to 5 (0 – no anticipated effect; 5 – maximum anticipated effect). Key: Facilitators – Organisational Vision^a^, Empowerment^b^, Collaboration^c^, Resources^d^; Barriers – Lack of resource^e^, Resistance to change^f^, Complexity^g^, Lack of leadership^h^.

The interviews identified 12 barriers to the design and delivery of sustainable healthcare in NHS Scotland (Fig. 1). After weighting, the strongest barriers were identified as lack of resources (4.8/5.0) and lack of whole systems and multi-disciplinary working (4.5/ 5.0). The barriers were categorised into four themes of: lack of resource; resistance to change; complexity; and lack of leadership.

1. Lack of resource

Lack of resource in terms of money, time, and staffing were identified as barriers to change. In the context of limited funding and cost-saving efforts, interviewees found it hard to make a case for the upfront financial expense of purchasing new ‘greener’ equipment or making changes to infrastructure to enable re-use of equipment (eg increasing capacity of laundry or sterilisation services). A lack of time was also cited, with many of our interviewees explaining that their sustainability efforts were rarely embedded into job roles and were instead conducted in their spare time. Alongside this extracurricular nature, interviewees explained that, amidst staff burnout, it was difficult to generate enthusiasm or capacity among colleagues to contemplate change. Interviewees reported very few instances of successful organisational communication which had been effective in involving and empowering staff in working towards the sustainability goals of their employing organisation.

Interviewees also discussed the lack of resources in the context of competing demands and acknowledged that more visible pressures such as treatment waiting times or bed shortages often diverted resource away from tackling the climate crisis which was deemed to be a less immediate threat. In addition, competing societal pressures such as the cost-of-living crisis made it difficult to gain consensus on sustainable healthcare being a priority area.

The overall perception of a lack of resource led to a feeling that, while high-level plans often set ambitious net-zero targets, it was very difficult to translate this into practice.

2. Resistance to change

Interviewees described how resistance to change was encountered among staff and infrastructures. Explanations for staff resistance included people lacking the confidence or competence to adopt new techniques or use new equipment; people being wary of the clinical effectiveness, safety and/or infection control implications of proposed sustainable alternatives; and people disagreeing that climate change was an issue that required action. A lack of clear organisational communication was felt to contribute to this issue.

Barriers to creating environments that were amenable to change were also discussed in the context of staff and systems that were operating under increasing strain. For example, it was felt that working at or close to full capacity resulted in a state of reactive working in which there was no space to consider new ways of doing things. There was also wariness of placing yet another burden upon the shoulders of an already stretched workforce, and awareness of the risk of creating climate anxiety.

3. Complexity

Complexity of both the planetary crisis as an issue and the healthcare system were identified as further barriers to change. Regarding the former, the volume of literature on the planetary crisis, a perceived lack of competence in conducting carbon calculations, and an absence of robust literature to evidence the impact of proposed changes were examples of complexities that were perceived to be difficult to overcome.

Within healthcare systems, sustainable healthcare changes were rarely being applied *de novo* but rather had to be incorporated into existing systems and infrastructures. Old buildings, procurement pathways, and governance structures were examples of complexities within the system that were difficult to navigate.

4. Lack of leadership

A lack of leadership was felt to increase the risk of silo working which arises when individuals, teams and/or organisations have no insight to other's work. This was felt to contribute to duplication of effort and prohibited transfer of knowledge and upscaling of new ideas. For sustainable healthcare to flourish, it was felt that organisations needed leaders who had systems-level oversight and who were willing to learn from and adopt good practice from elsewhere.

A lack of leadership was also identified as a barrier to achieving long-term change. For example, staff in rotational posts often felt that promising work failed to progress when there was a lack of leadership among permanent staff to support successful short-term pilots being embedded into routine practice.

### The facilitators to the design and delivery of sustainable healthcare

The interviews identified 12 facilitators to the design and delivery of sustainable healthcare in NHS Scotland (Fig. 1). After weighting, the strongest facilitators were identified as ‘effective leadership and culture of empowerment’ (4.8/5.0) and ‘whole systems and multi-disciplinary working’ (4.8/5.0). The facilitators were categorised into four themes: organisational vision; empowerment; resources; and collaboration.

1. Organisational vision

Interviewees described several facilitators that arose when clear visions to achieve net-zero targets were declared. At an international level, interviewees cited the impact that COP-26 had on moving sustainability up the priority list, when over 50 countries made commitments to build climate resilient and low-carbon health systems. At a national level, Scotland's vision for net-zero healthcare by 2040 and subsequent strategy were seen as key in making healthcare organisations act on the climate crisis. At a local level, clear organisational visions were perceived to have led to the development of action plans; given permission to interested individuals to pursue sustainable change; motivated wider staff groups to get involved; and resulted in greater resource allocation. This was felt to have been particularly important for accelerating the impact of grassroots work, as it became easier and faster for small groups of individuals to get senior leadership support for their efforts when work aligned with overarching organisational priorities.

2. Empowerment

Within the theme of empowerment, the first facilitator described was that of personal values. Several interviewees described how their interest in the planetary crisis in their personal lives had empowered them to consider and take action to address the environmental impact they were having within their professional roles. In turn, these individuals were seen to empower others to follow their lead. This was coupled by the empowering impact that visible and impactful small effective changes in the early stages of work could have in sparking interest among colleagues.

A further facilitator that fell into the theme of empowerment was the value of building networks of like-minded individuals. Networks were cited as sources of support, motivation, and solutions when barriers to change were being met and as sources of shared learning and new ideas.

3. Resources

While lack of resources was identified as a barrier, the opposite was recognised as a facilitating theme. In the first instance, when staff had sustainability incorporated into their roles and remits, this enabled them to put aside time to push forward with sustainable change, which was felt to significantly accelerate progress.

Secondly, educational resources were identified as equipping staff with an understanding of why sustainable action was required, and the knowledge of what actions they could take to contribute to net-zero ambitions. Supporting the translation of theory into practical application, day-to-day resources such as ‘green’ toolkits and checklists were felt to facilitate sustainable healthcare becoming the default option across all staff groups.

Finally, interviewees discussed how the co-benefits of sustainable healthcare could often lead to a gain in resources. For example, reducing wasteful practice and optimising disease management were highlighted as not only reducing environmental harm through a reduction in healthcare use but, in turn, also saving staff time and money and reducing patient morbidity.

4. Collaboration

All interviewees mentioned the importance of partnership working in achieving sustainable change. Collaborations were cited within single professional groups, such as networks of general practitioners or anaesthetists, but also between multiple professional groups, such as Green Theatres initiatives which drew upon multi-disciplinary input. In addition, collaborations were discussed across the wider system. Some interviewees cited the UK Health Alliance on Climate Change as an example of bringing many different health organisations under one banner to promote sustainable healthcare, whilst others referenced the importance of multiple sectors such as academia, pharmaceuticals, and healthcare all having to work together to achieve net-zero healthcare. Finally, patients were identified as key partners in achieving change, and some interviewees gave examples of patients being the ones to initiate conversations with healthcare professionals about the environmental impact of their care and how to minimise this.

## Discussion

### Summary of findings

This forcefield analysis exercise identified 12 facilitators and 12 barriers to the design and delivery for sustainable healthcare across NHS Scotland. To overcome the barriers and strengthen the facilitators, action is required across all levels of NHS Scotland, from individuals through to whole systems.

The findings highlight that creating a vision for environmentally sustainable healthcare is key to focusing priorities and allocating resources to achieve this aim. With a vision in place, it is then essential to empower people to get involved. Empowerment is permitting and facilitating staff to apply their personal sustainability values to their professional life, and the subsequent enthusiasm that this generates can inspire others to join. Combining a range of knowledge, resources and expertise is essential to bring about effective change, highlighting the importance of collaboration between professional groups and organisations in efforts to achieve sustainable healthcare.

In opposition of the facilitators, there are barriers that impede efforts to achieve sustainable healthcare. In the first instance, scarce resources and competing demands inhibit timely progress towards net-zero. Furthermore, resistance across the system also slows progress as individuals and teams can be wary of changing their practice due to a lack of confidence or competence in new methods or devices. Further upstream, resistance is encountered in policies or infrastructures that require intensive resource to change. The difficulty in obtaining robust data to evidence impactful change adds to the perceived complexity of taking action against climate change and requires addressing.

### Strengths and limitations

To the authors’ knowledge, this is the first report to describe the barriers and facilitators to sustainable change in NHS Scotland. The findings reflect a range of stakeholders from multiple professional groups, at differing levels of seniority, and who work in different geographical areas across Scotland. As sampling continued until saturation was reached, it is felt that the findings offer a comprehensive overview of the barriers and facilitators being encountered. The study authors were also from a range of clinical backgrounds and took a collaborative approach to data collection and analysis, minimising the introduction of personal biases in results interpretation.

Considering the study limitations, the results were based upon a relatively small sample size and, due to the nature of those interviewed, may be biased towards the views of NHS staff who have an active interest and involvement in sustainable healthcare. To overcome this, an area of future work would be to pose the same questions to a larger sample of NHS Scotland staff and to include a sample of both those with and without an interest and/or involvement in sustainable healthcare. The validity of the weighting component could also be strengthened by wider sampling.

### Implications for future practice

To complete the final step of Lewin's forcefield analysis, ten recommendations (Table 1) were formulated by the study group which could help to overcome barriers and strengthen facilitators so that the likelihood achieving sustainable change is increased.

**Table 1:** Recommendations to overcome barriers and strengthen facilitators to the design and delivery of sustainable healthcare.

1	Communicate with the general public, NHS staff, and wider system partners on NHS Scotland's organisational vision for net-zero healthcare and the leading role each can play in attaining this ambition.
2	Organisations should make net-zero a priority with clear action plans, leadership, and accountability to accompany this.
3	Create a culture of empowerment that enables leadership among those with a climate interest.
4	Allocate human, economic and time resources to sustainable change efforts.
5	Consider environmental sustainability in all healthcare planning and procurement decisions.
6	Make carbon footprint calculations more accessible.
7	Develop a national repository of carbon footprint metrics.
8	Create and strengthen networks to facilitate shared learning, resources and good practice.
9	Promote multi-disciplinary and cross-organisational working.
10	Work with academics and industry to harness innovation.

As illustrated by the scope of recommendations, for NHS Scotland or any other healthcare service to achieve net-zero, a comprehensive programme of activities that span across individuals, teams, organisations, and systems will be required. There is, therefore, a role for everyone who is involved in the design and/or delivery of healthcare to play their part.

## Conclusion

This study demonstrates that change management tools such as forcefield analyses can be used to identify the barriers and facilitators to sustainable healthcare. By gaining foresight of these influences, action can be taken to weaken the former and strengthen the latter, which may increase the likelihood of successful change. As the barriers and facilitators identified in this study are likely to resonate with sustainable healthcare efforts elsewhere in the UK and internationally, application of the proposed recommendations may increase the likelihood of these efforts being successful, whilst repetition may uncover further facilitators, barriers and actions that are unique to other contexts.

## Disclaimer

This article reflects the opinions of the author(s) and should not be taken to represent the policy of the Royal College of Physicians unless specifically stated.

## Funding sources

This research did not receive any specific grant from funding agencies in the public, commercial, or not-for-profit sectors.

## Interviewee consent

All interviewees and contributors gave expressed consent for their answers and opinions to be reflected in this study and academically published, on an anonymous basis.

## Author contributions

AH, KC, ET, KJ, LH, RT and PF interviewed study participants. All authors contributed equally to thematic analysis and forcefield scoring. All authors contributed to manuscript preparation.

## Declaration of competing interest

The authors declare that they have no known competing financial interests or personal relationships that could have appeared to influence the work reported in this paper.
